# LAMP Assay for the Detection of *Echinococcus* *multilocularis* Eggs Isolated from Canine Faeces by a Cost-Effective NaOH-Based DNA Extraction Method

**DOI:** 10.3390/pathogens10070847

**Published:** 2021-07-05

**Authors:** Barbara J. Bucher, Gillian Muchaamba, Tim Kamber, Philipp A. Kronenberg, Kubanychbek K. Abdykerimov, Myktybek Isaev, Peter Deplazes, Cristian A. Alvarez Rojas

**Affiliations:** 1 Institute of Parasitology, Vetsuisse and Medical Faculty, University of Zurich, 8057 Zurich, Switzerland; barbara.bucher@uzh.ch (B.J.B.); gillian.muchaamba@uzh.ch (G.M.); timpeter.kamber@uzh.ch (T.K.); philipp.kronenberg2@uzh.ch (P.A.K.); deplazesp@access.uzh.ch (P.D.); 2 Graduate School for Cellular and Biomedical Sciences, University of Bern, 3012 Bern, Switzerland; 3 Section of Epidemiology, Vetsuisse Faculty, University of Zurich, 8057 Zurich, Switzerland; kubanychbek.abdykerimov@uzh.ch; 4 Life Science Zurich Graduate School, University of Zurich, 8057 Zurich, Switzerland; 5 Department of Parasitology, Kyrgyz Research Institute of Veterinary Medicine Arstanbek Duisheev, Ministry of Education and Science of the Kyrgyz Republic, Bishkek 720033, Kyrgyzstan; isaev-ww-1988@mail.ru

**Keywords:** *Echinococcus*, NaOH, LAMP, PCR, DNA extraction, taeniid egg isolation

## Abstract

The detection of *Echinococcus multilocularis* in infected canids and the environment is pivotal for a better understanding of the epidemiology of alveolar echinococcosis in endemic areas. Necropsy/sedimentation and counting technique remain the gold standard for the detection of canid infection. PCR-based detection methods have shown high sensitivity and specificity, but they have been hardly used in large scale prevalence studies. Loop-mediated isothermal amplification (LAMP) is a fast and simple method to detect DNA with a high sensitivity and specificity, having the potential for field-application. A specific LAMP assay for the detection of *E*. *multilocularis* was developed targeting the mitochondrial *nad1* gene. A crucial step for amplification-based detection methods is DNA extraction, usually achieved utilising silica-gel membrane spin columns from commercial kits which are expensive. We propose two cost-effective and straightforward methods for DNA extraction, using NaOH (method 1A) and InstaGene^TM^ Matrix (method 1B), from isolated eggs circumventing the need for commercial kits. The sensitivity of both assays with fox samples was similar (72.7%) with multiplex-PCR using protocol 1A and LAMP using protocol 1B. Sensitivity increased up to 100% when testing faeces from 12 foxes infected with more than 100 intestinal stages of *E*. *multilocularis*. For dogs, sensitivity was similar (95.4%) for LAMP and multiplex-PCR using protocol 1B and for both methods when DNA was extracted using protocol 1A (90.9%). The DNA extraction methods used here are fast, cheap, and do not require a DNA purification step, making them suitable for field studies in low-income countries for the prevalence study of *E*. *multilocularis*.

## 1. Introduction

*Echinococcus multilocularis* is the canine intestinal cestode responsible for alveolar echinococcosis (AE) in humans [1]. Primarily foxes, and to a lesser extent, other wild canids and domestic dogs play a role as definitive hosts for *E*. *multilocularis* [2]. Humans act as dead-end hosts, acquiring the infection by oral intake of viable eggs of *E*. *multilocularis* from infected food, soil, water, or hand-to-mouth contact after a direct interaction with infected dogs and contaminated matrices [3]. Establishing the exact link between AE and its origin (source attribution) is challenging due to the long incubation time of the disease of up to 15 years. Therefore, it is essential to have tools for the accurate determination of the environmental contamination with *E*. *multilocularis* eggs and the detection of canine intestinal infections.

Different methodologies for the diagnosis of *E*. *multilocularis* in canids have been standardised and published over the years. Post-mortem examination (i.e., sedimentation and counting technique, SCT) remains the gold standard [4]. However, a post-mortem examination has significant disadvantages, requiring either expensive infrastructure, strict biosafety measures and involves the killing of canids. Several methods based on the detection of *E*. *multilocularis* antigens [5,6,7,8] and DNA [9,10,11] in faeces have been developed (reviewed in [12,13]). Coproantigen detection (copro-ELISA) has the advantage of detecting prepatent infections [5,14]. Although some coproantigen tests are commercially available, they are not regularly used for massive screening in canid faeces from endemic areas. On the other hand, DNA of *E*. *multilocularis* can be potentially detected from the prepatent period as free DNA or from cells of the growing worms; and mostly from proglottids/eggs during patency. DNA can be isolated from total faeces and used, for example, in nested PCR (copro-DNA-PCR) [9] or from isolated taeniid eggs through floatation and sieving [10] and used in multiplex-PCR (egg-DNA-PCR) [15]. Although the egg-DNA-PCR focuses only on patent infections, it has the advantage of concentrating eggs and decreasing the presence of PCR inhibitors.

PCR-based methods for identifying parasites in faecal samples rely on isolation and DNA concentration using silica-gel membrane in spin columns from commercial kits removing PCR inhibitors (proteinases, bile salts, polyphenols, and acids). However, some publications report that the total removal of inhibitors from faeces is not always possible [16,17]. Furthermore, the usage of commercial kits requires trained staff performing several liquid handling steps, and the commercial kit itself, which can be a costly item when large numbers of samples need to be analysed. The cost of such kits ranges between 3 and 11 USD per sample, which is unreasonable for governments and researchers in low-income countries. Besides the PCR inhibitors, the successful disruption of the typical taeniid eggshell keratin-like layer by homogenisation or alkaline lysis is essential for DNA detection. Unlike polymerases used in PCR, the in silico designed *Bst* 2.0 DNA Polymerase used in LAMP (loop-mediated isothermal amplification) displays improved tolerance for inhibitors [18,19,20]. LAMP assays have been developed to detect *E*. *multilocularis* [21,22] and *E*. *granulosus* [23,24,25,26] in faecal samples and also several other parasites, including *Clonorchis sinensis* [27], *Trichuris muris* [28], *Strongyloides* spp. [29], *Necator americanus* [30], and *Taenia* spp. [31]. LAMP also offers a robust tool for DNA amplification on the presence of reagents like NaOH used in alternative DNA extraction methods suggesting that commercial kits for DNA isolation could be circumvented. Methods for DNA extraction without silica-gel membranes have been described in the literature since the 1990s [32,33,34,35]. A reappraisal of such methods for developing cost-effective DNA extraction (i.e., NaOH-based) has provided evidence to produce a template of sufficient quality for subsequent PCR from different tissues [36,37,38]. Furthermore, single *Echinococcus* eggs have been used for PCR- and LAMP-based methods directly after treatment with NaOH [26,39,40,41,42]. In the present study, we propose two cost-effective DNA extraction methods (without purification) from isolated taeniid eggs, based on NaOH and InstaGene^TM^ Matrix (Chelex 6%) to be used directly as a template for multiplex-PCR [15] and LAMP (developed in this study) for the detection of *E*. *multilocularis*. We also labelled two of the primers used in LAMP to combine them with lateral flow dipstick for the same diagnostic purpose. The proposed cost-effective DNA extractions can be used in large prevalence studies to detect *E*. *multilocularis* eggs and potentially *E*. *granulosus* in canine faecal samples in low-income endemic countries.

## 2. Results

### 2.1. Optimisation, Specificity, Analytical Sensitivity, Limit of Detection, and Stability of LAMP

After optimisation of the LAMP assay, the reaction was set up with 6 mM MgSO_4_, 0.8 M betaine, 0.004% malachite green, and 1× primer mix (1.6 µM of each FIP and BIP primer, 0.2 µM of each F3 and B3 primer and 0.4 µM of the loop primer); amplification occurred at 65 °C for 60 min. The selected LAMP primer set showed successful amplification when using *E. multilocularis* DNA rendering a clear blue-green colour, a ladder-like structure on agarose gel after electrophoresis and a specific band on the lateral-flow-dipstick (when using the labelled BIP and FIP primers). Conversely, a negative result followed by testing DNA of other *Echinococcus* spp., *Taenia* spp., and other cestodes as a template (see material and methods). The limit of detection (analytical sensitivity) of LAMP with the selected primer set was 1 pg/µL of *E. multilocularis* DNA in all serial dilutions performed in triplicate. On the other hand, the limit of detection of the multiplex-PCR [15] was 10 fg/µL of *E*. *multilocularis* DNA. Serial dilutions of samples derived from protocol 1A were performed to exclude inhibition of LAMP reactions. The 1:50 dilution showed to perform best, as no inhibition could be detected. The lowest amount of eggs possible to detect with LAMP and multiplex-PCR was one egg treated with the protocol 1B in 4/4 replicates and two eggs in 3/4 replicates with protocol 1A. In order to investigate if free DNA of the parasite was present in the supernatant of the egg suspensions used for the limit of detection we used it as a template for LAMP and multiplex-PCR [15], all reactions were negative. In this way, we can confirm that DNA originated from the eggs in suspension. Aliquots of a prepared master mix for LAMP containing all the reagents except DNA were tested weekly over six weeks. A positive LAMP reaction was achieved in all the period when adding *E*. *multilocularis* DNA (2 ng).

### 2.2. Examination of Field Samples with Protocols 1A and 1B

**Figure 1:**
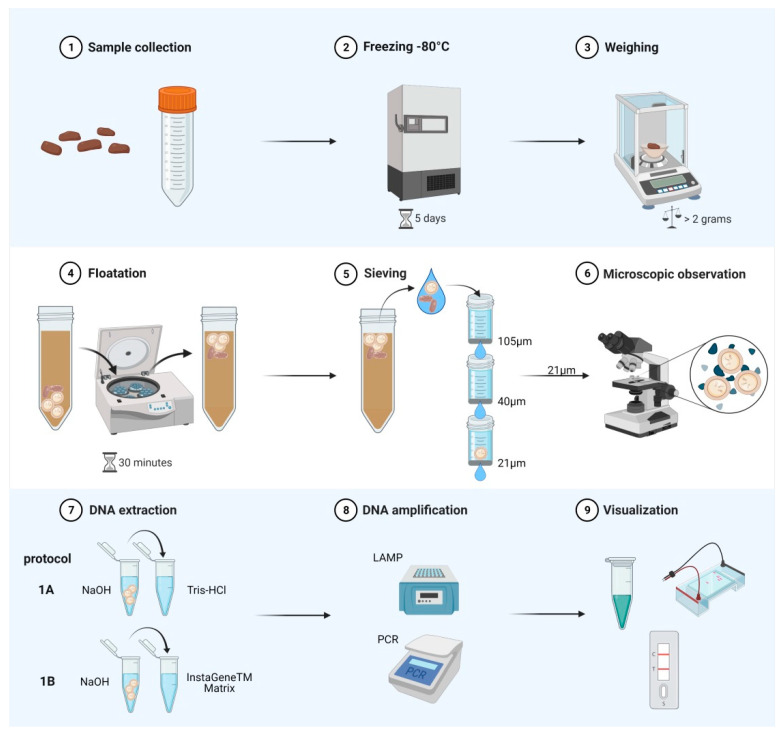
Schematic representation of the process of field samples analysed in the present study. Step 7 shows the two methods used for DNA extraction in this study (protocols 1A and 1B). The visualisation of multiplex-PCR occurred after electrophoresis and in the case of LAMP results were assessed after change of colour of the reaction, electrophoresis, and lateral flow dipsticks as shown in step 9.

A schematic representation of the sample processing is shown in Figure 1. In the case of negative samples, we collected the sediment from the 21 µm filter, in the same way as in positive samples, to perform the DNA extraction.

#### 2.2.1. Foxes

Intestinal stages of *E. multilocularis* (between 2 to >100) were detected in 44/62 foxes after necropsy/SCT. Microscopic detection of taeniid eggs was successful for 30 samples from the 44 foxes documented to be infected with *E*. *multilocularis*. On the other hand, 18 faecal samples were collected from foxes without intestinal stages of *E*. *multilocularis* at necropsy/SCT; from them, it was possible to observe taeniid eggs in five samples (Table 1); 8/18 foxes mentioned before were infected with *Taenia* spp. When using protocol 1A, LAMP showed a positive reaction in 26/44 samples from animals harbouring *E*. *multilocularis* in their intestine and 1/18 samples from foxes without intestinal stages of *E*. *multilocularis*. Conversely, multiplex-PCR was positive for *E*. *multilocularis* in 32/44 samples from foxes infected with *E*. *multilocularis* and 1/18 samples from foxes without intestinal stages of *E*. *multilocularis*. Using multiplex-PCR, other cestodes and *Taenia* spp. were identified, producing an amplicon (267 bp) in five samples from the 18 animals without intestinal stages of *E. multilocularis* (protocol 1A and 1B), suggesting that if PCR inhibitors were present, they did not impede the amplification of DNA (See detailed list of results in Table S1).

The second aliquot of faeces from the same foxes was used to isolate eggs for treatment with protocol 1B. In this case, taeniid eggs were observed in 31/44 faeces from animals with *E*. *multilocularis* in their intestine and in 5/18 faecal samples from negative animals. After treating eggs with protocol 1B, we found positive LAMP reactions in 32/44 foxes infected with *E*. *multilocularis* and in 1/18 foxes with no intestinal stages of *E*. *multilocularis*. Conversely, multiplex-PCR was positive for *E*. *multilocularis* in 30/44 positive and 1/18 negative foxes, respectively. A positive result for the amplification of 267 bp corresponding to other cestodes including *Taenia* spp. was found in 10 samples with a negative result for *E. multilocularis* using the DNA from protocol 1B (Table S1).

Furthermore, multiplex-PCR using protocols 1A and 1B showed a positive result for the amplification of 267 bp corresponding to other cestodes, including *Taenia* spp. in all the eight foxes identified to be infected with *Taenia* spp. at necropsy/SCT.

#### 2.2.2. Dogs

The detection of *E*. *multilocularis* in dogs was performed by a multiplex-PCR in a previous prevalence study. In the case of the dog group (Table 1), taeniid eggs were observed in 20/22 samples from dogs previously identified to be infected with *E*. *multilocularis*. No eggs were observed in samples that were considered negative to *E*. *multilocularis*. When the isolated eggs were treated with protocol 1A and used for DNA amplification, we found a positive result for *E*. *multilocularis* in 20/22 samples using LAMP and multiplex-PCR. No positive reactions occurred with LAMP and multiplex-PCR in the ten samples previously identified as negative to *E*. *multilocularis*. When isolating taeniid eggs to be treated with protocol 1B, we observed eggs in 21/22 samples from dogs previously identified positive to *E*. *multilocularis*. In this group, LAMP and multiplex-PCR showed a positive result in 21/22 samples and a negative result in 10/10 samples identified as negative to *E*. *multilocularis.*

In the case of fox samples, sensitivity in LAMP and multiplex-PCR was 72.7% (C.I. 95%: 57.2–85) using protocol 1B and 1A, respectively (Table 2). Specificity values were not 100% for fox samples, the highest value was 94.4% (72.7–99.8) achieved using both protocols for LAMP and multiplex-PCR (Table 2). In the dog group, sensitivity for LAMP and multiplex-PCR was 95.4% (77.1–99.8) achieved with protocol 1B. The specificity in LAMP and multiplex-PCR with both protocols was 100% (Table 2).

### 2.3. Examination of Field Samples with Protocols 1A and 1B According to Worm Burden

From 28 fox samples (positive at necropsy/SCT) collected in 2020, we assessed the worm burden for *E. multilocularis* after a thorough examination of the intestinal mucosa. Worm burden ranged between 2 and >100 *E. multilocularis* intestinal stages (Table 3). We divided the samples based on worm burden from foxes with two to 20 intestinal stages of *E. multilocularis* (9 foxes), 21–100 (7) and samples from foxes with >100 worms (12). We also included 17 samples from foxes that were negative at necropsy/SCT for *E. multilocularis*. LAMP and multiplex-PCR showed a positive result in 1/9 samples with worm burden between two and 20 parasites. When worm burden was between 21–100 worms, LAMP could detect 4/7 of the samples with protocol 1A and 6/7 were positive with multiplex-PCR. With protocol 1B, LAMP detected 6/7, and multiplex-PCR found 7/7 to be positive. Finally, when the worm burden was higher than 100 worms LAMP and multiplex-PCR detected 12/12 positive samples with protocol 1B. Multiplex-PCR detected 12/12 samples positives with protocol 1A (Table 3). Finally, from the 17 samples with no *E. multilocularis* found at necropsy/SCT, LAMP and multiplex-PCR rendered a positive result in one sample treated with both protocols.

### 2.4. DNA Extraction from Whole Faeces (Protocol 2)

We investigated if it was possible to use NaOH+InstaGene^TM^ Matrix (called protocol 2A) to treat a faecal sample to be used directly as a template for LAMP reaction (from this study) and a multiplex-PCR [15]. For comparison, we isolated DNA from the same samples using the Qiagen mini stool kit (named here protocol 2B). Using protocol 2A, we found a positive LAMP reaction for *E. multilocularis* in 10/30 faeces from foxes positive to *E. multilocularis* at necropsy/SCT; and a positive result in 17/30 in multiplex-PCR (Table 4). When an aliquot of the same samples was used for DNA isolation with the commercial kit, LAMP was positive in 16/30 and multiplex-PCR in 20/30 samples. Furthermore, a positive amplification corresponding to other cestodes, including *Taenia* spp. [15] was found in seven and five samples which were negative to *E. multilocularis* using a template from protocol 2A and the QIAGEN stool kit, respectively, with the multiplex-PCR. Detailed record of the multiplex-PCR results showing amplification of other cestodes, including *Taenia* spp. can be found in Table S1.

In the case of dogs, LAMP was positive in 2/18 samples and 7/18 samples in multiplex-PCR with protocol 2A. Using DNA isolated with a commercial kit, we found a positive LAMP reaction in 7/18 samples and 6/18 using the multiplex-PCR. Overall, the sensitivity achieved with the multiplex PCR using protocol 2B was 66% (CI: 47.1–82.7) followed by multiplex-PCR using protocol 2A (56.6%, 37.4–74.5) both in fox faeces. For dog faeces, the sensitivity achieved was 38.8% (17.3–64.2) with multiplex-PCR using protocol 2A and LAMP using protocol 2B.

## 3. Discussion

Loop-mediated isothermal amplification (LAMP) [43] has been portrayed as an affordable alternative to PCR for the detection of different pathogens, including parasites in faeces [18,19,44,45]. *Bst* polymerase used in LAMP offers high robustness being able to withstand harsh chemicals like NaOH and to overcome the presence of PCR inhibitors. In this study, we used the *Bst* 2.0 DNA polymerase which has improved performances regarding amplification speed, yield, tolerance to salt, and inhibitors present in different matrices including faeces [18,19,20]. Nevertheless, in the present study, a dilution of samples was necessary for protocol 1A, either to dilute NaOH or inhibitors present in samples. We developed a simple, cost-efficient methodology for DNA extraction involving lysis of isolated taeniid eggs with NaOH alone or adding Chelex resin (InstaGene^TM^ Matrix). Then, we coupled the DNA extraction with a LAMP test developed in this study. Additionally, we included the multiplex-PCR [15] to compare the results for the amplification of the *E*. *multilocularis* target.

Necropsy/SCT remains the gold standard for the detection of *Echinococcus* spp. in canids but it is not rational to perform. We had access to freshly hunted foxes from which a necropsy and SCT could be performed. Therefore, we were able to compare the performance of the methods for DNA extraction using LAMP and multiplex-PCR [15] to detect *E*. *multilocularis* in fox faecal samples against the gold standard. As seen in Table 2**,** the sensitivity of both assays with fox samples was 72.7 (57.2–85) with multiplex-PCR using protocol 1A and LAMP using protocol 1B. We performed a thorough worm count in the intestinal content of foxes necropsied in 2020, showing that the sensitivity of the tests increased up to 100% in multiplex-PCR using protocol 1A for DNA extraction in faeces from 12 foxes with more than 100 worms. Protocol 1B LAMP and multiplex-PCR also detected 12/12 positive samples. It is possible to suggest that sensitivity could be improved further using a higher amount of faeces as starting material to increase the chances of finding eggs in animals with low worm burden.

In samples from dogs, the infection status was assessed with multiplex-PCR from isolated eggs from faeces collected from the ground in a highly endemic area for AE in Kyrgyzstan. In this case, sensitivity was 95.4% for LAMP and multiplex-PCR using protocol 1B (Table 2) and 90.9% for both methods when DNA was extracted using protocol 1A. In the present study, we were able to detect other cestodes, including *Taenia* spp. and *Mesocestoides* spp. in some of the samples which were negative for *E*. *multilocularis* using the multiplex PCR developed by Trachsel et al. [15], suggesting that no inhibition was present in these samples. Furthermore, we detected *Taenia* in all the samples from naturally infected foxes harbouring adult *Taenia* specimens in their intestine. By using whole faeces (400–500 mg) as starting material, multiplex-PCR was more sensitive than LAMP detecting *E*. *multilocularis* infection in foxes and dogs that using DNA isolated with a commercial kit. We used a large amount of faecal sample (500 mg) as input for the QIAamp FAST DNA Stool Mini Kit. Using the same commercial kit, Skrzypek et al. [46] used 1 g of faeces to detect *E*. *multilocularis* reporting positive results in 45.7% and 48.6% of the faeces from infected foxes (diagnosed at necropsy/SCT) with nested and multiplex-PCR, respectively, which is similar to the results found by us.

Previously, a LAMP assay has been used to detect *E*. *multilocularis* in canine faeces in China, reporting higher sensitivity than PCR in experimentally and naturally infected dogs, detecting as low as 1 pg DNA [21] which is the same limit of detection in the LAMP from the present study. The LAMP assay developed by Ni et al. has been used for detecting *E*. *multilocularis* [21] in wastewater in China [47]. Interestingly, in the work by Ni et al. [48], DNA was isolated with the QIAamp DNA Stool Mini Kit which in our hands did not produce satisfactory results. However, the results using the commercial kit were better than using NaOH-based method directly in faeces. The same kit was used in another report of a LAMP assay for the detection of *E*. *multilocularis* and other taeniids in a Tibetan rural area [22]. The LAMP assay detected 1 pg of DNA from *E*. *multilocularis* and a minimum of two eggs from this parasite [22]. They found that multiplex-PCR was able to detect more samples infected with *E*. *granulosus* and *T*. *hydatigena* than LAMP.

Finally, LAMP has been portrayed as a simple and easy to implement method that could be used in field surveillance of pathogens, specifically for *Echinococcus* species. However, so far it has not been used in large epidemiological studies in endemic areas. One of the advantages of LAMP is that there is no need for a thermocycler; however, it still requires some equipment including a heating block or water bath, a centrifuge, gel electrophoresis system (if results need to be confirmed in case of ambiguous colour change), and a clean lab with trained staff. In LAMP reactions, large quantities of DNA are amplified and pose a high risk of contamination when tubes are opened. To minimise this risk, a typical lab where PCR is routinely used, needs different separate environments for DNA extraction (pre-LAMP), setup of LAMP reactions, and visualisation of LAMP results (post-LAMP). The irregular shedding of eggs by the definitive hosts hampers the correct diagnosis of patent *E*. *multilocularis* infections. Furthermore, there are differences in the egg shedding between hosts; the highest egg output occurs 37 to 42 days post experimental infection in foxes and between 43 and 45 days in dogs in the same conditions [49]. Additionally, the patent period lasts one month for 98% of the worm burden, with a residual worm burden that lasts several months [49]. Therefore, detecting eggs from naturally infected canids in a single sample from naturally infected animals is challenging, as we have shown here since the presence of eggs will depend on the stage of the infection. Furthermore, the taeniid egg isolation has some disadvantages which is important to keep in mind, it can be time consuming especially analysing large number of samples, it requires the purchase of nylon filters which need to be inserted in the lid of the tubes or PET bottles [50] for sieving and it involves the use of a floatation solution which in the case of zinc chloride can be toxic for the manipulator and the environment [51]. A sugar solution [52] could potentially replace the zinc chloride avoiding the toxicity. We also tested the feasibility of using lateral flow dipstick coupled with LAMP reaction, and the results are promising; however, the high cost of the dipsticks precluded us from suggesting them as a cost-effective tool for the diagnosis of *E*. *multilocularis*.

Methods based on DNA detection from *E*. *multilocularis* have been published and extensively reviewed [12,13,53]. In general, they can be divided into those using whole faeces and the ones based on isolated taeniid eggs as starting material for DNA extraction. We focus in this study on the latter method which allows the enrichment of helminth eggs from faecal samples; in doing so, we aim to decrease the presence of PCR inhibitors commonly found in faecal samples based on the thorough wash of the filters used in the sieving process [10,15]. The taeniid egg isolation based on floatation/sieving allows the use of a relatively large amount of faeces as starting material, increasing the chances of finding eggs in canines with low worm burden for example. Up to 20 mL of faeces suspended in ethanol were used in the original publication describing the method [10] reporting a positive PCR in 33 out of 35 samples from foxes infected with *E*. *multilocularis* (diagnosed at necropsy). On the other hand, protocols for DNA extraction from whole faeces using commercial kits generally accept from 100 up to 500 mg of faeces as starting material and have the advantage of acquiring high yield purified DNA, in theory without PCR inhibitors. But they are expensive, costing between 3.1 and 10.2 USD per sample, and require between 40 to 90 min to be completed for 10 samples [16], making them unsuitable for use in large-scale prevalence studies. Therefore, there is a need to investigate and standardise cost-effective and straightforward methods for DNA extraction to be used in such studies. The use of NaOH-based DNA extraction methods from different tissues can produce a template of sufficient quality for PCR [36,37,38,54]. Furthermore, DNA has been extracted from *Echinococcus* spp. eggs, and also protoscoleces and cyst tissue, using NaOH for direct PCR and LAMP for genotyping studies without a purification step in several publications [26,39,40,41,55,56]. Similarly, the use of Chelex as a DNA extraction method has also been proposed since the 1990s [33] and used in recent publications as an alternative to producing input for PCR-based diagnostics [57,58] and mammal identification of scats [58] for example. NaOH and Chelex are components of the method used in seminal publications reporting on DNA isolation from taeniid eggs in which a commercial kit was also included [10,11,15,59]. The DNA extraction methods used in the present study offer a cost-effective alternative to the commercial kits for this purpose. If we consider the cost of a NaOH 0.2 M commercially available, the cost per sample is 0.014 USD for protocol 1A; in the case of protocol 1B the cost of InstaGene^TM^ Matrix per sample is 0.95 USD. Furthermore, the time of DNA extraction is reduced in protocol 1A to 20 and 40 min with protocol 1B for processing ten samples, offering advantages over the 90 min required for DNA extraction described [59]. However, the time needed to isolate eggs needs to be considered as 3 h are required for 10–20 samples (Figure 1).

To conclude, we show two cost-effective methods for DNA extraction from isolated taeniid eggs for direct use in multiplex-PCR and LAMP developed in this study. These procedures circumvent the use of commercial kits for DNA extraction. Compared with the gold standard, the sensitivity of the test remains lower mainly because our methodology is focusing on the detection of eggs, therefore being unable to detect prepatent or low worm burden infections. Nevertheless, considering the reduction in costs and time, we propose the methods for DNA extraction as a valuable tool that can be used in extensive prevalence studies investigating the presence of eggs in canine environmental faecal samples in endemic areas of *E*. *multilocularis*.

## 4. Materials and Methods

### 4.1. Parasites

Eggs of *E*. *multilocularis* were isolated from the faeces of a naturally infected fox with no *Taenia* or *Mesocestoides* spp., detected at necropsy. Faeces were stored at −80 °C for five days for biosafety reasons and then subjected to floatation with zinc chloride (specific gravity 1.45) and sieving protocol with different nylon filters [10]. Eggs were stored at 4 °C in PBS with penicillin-streptomycin until further use. Multiplex-PCR [15] confirmed that only eggs of *E*. *multilocularis* and no *Taenia* spp. or *Mesocestoides* spp. were present in the suspension.

DNA was isolated from *E*. *multilocularis* adult worms and metacestode tissue cultured in vitro [60] using the QIAamp DNA Mini Kit (Qiagen, Hilden, Germany). The same procedure was used to isolate DNA from metacestode or adult stages of different cestodes identified morphologically including *E. granulosus sensu stricto*, *E. equinus*, *E. ortleppi*, *E. intermedius* (G6 and G7), *E*. *vogeli*, *E*. *shiquicus*, *Taenia polyacantha, T. multiceps, T. ovis, T. saginata, T. solium, T. crassiceps, T. hydatigena, Hydatigera taeniaeformis, T. pisiformis, T. krabbei, Dipylidium caninum, Diphyllobothrium latum, Mesocestoides litteratus*, and *M. lineatus*. Parasite identification was confirmed with PCR/sequencing of a section of the *cox1* gene [61].

### 4.2. LAMP

#### 4.2.1. Primer Design

Multiple alignments of the mitochondrial genome of *E*. *multilocularis* and other *Echinococcus* and *Taenia* species were performed to select a region for designing LAMP primers using Genious R10 V10.1.3. The mitochondrial *nad1* gene of *E. multilocularis* (accession number in GenBank AB668376) was selected as it showed sequence variation between species, and primers were created using Primer Explorer V5 (EikenChemicalCoLtd.) [62] and Primer Designer 1.16 (Premier) [63]. Primers were tested in silico for specificity using BLAST [64] and Primer-BLAST [65]. In total, seven primer sets, four primers each, were synthesised by Microsynth (Balgach, Switzerland). Primer sets were tested, assessing their specificity and analytical sensitivity to detect DNA from *E*. *multilocularis*.

#### 4.2.2. Specificity and Analytical Sensitivity

Initially, the primer sets were tested in LAMP reactions using DNA isolated from *E*. *multilocularis*. Serial dilutions (1:10) of *E*. *multilocularis* DNA were prepared in triplicate starting at 10 ng/µL to 0.1 fg/µL. The primer set showing the highest consistency, specifically amplifying only *E*. *multilocularis* DNA with the lowest concentration as a template, was chosen for standardisation of the LAMP reaction (Table 5). Additionally, we tested all primer sets with DNA from the different cestodes mentioned above. To allow the visualization of samples positive to *E*. *multilocularis* DNA with the HybriDetect-Universal Lateral Flow Assay Kit (Milenia Biotec, Giessen, Germany), the FIP and BIP primers were labelled at the 5′ end with FAM (6-fluorescein amidite) and DIG (digoxigenin) as shown in Table 5.

#### 4.2.3. LAMP Assay

For the optimisation of LAMP assays, different concentrations of MgSO_4_ (4–8 mM); primer mix (2×, 1.5×, 1×, 0.75×, 0.5×, 0.25×); betaine (0.4, 0.8, 1 and 1.2 M); malachite green (0.004%, 0.008% and 0.016%) were tested. Bovine serum albumin (0.1% w/v) was added to the reaction mixture to improve the performance of the *Bst* Polymerase during amplification. Various amplification times (30–90 min) and temperatures (61–70 °C) were tested. The reagent concentrations showing the strongest amplification (see the visualisation of the LAMP reaction) without unspecific amplification were chosen for the field study. Finally, the LAMP reaction was set up in 25 µL containing the isothermal amplification buffer from New England Biolabs (Ipswich, MA, USA) [20 mM Tris-HCl, 10 mM (NH_4_)_2_SO_4_, 50 mM KCl, 2 mM MgSO_4_, and 0.1% Tween 20], 6 mM MgSO_4_, 1.4 mM of each dNTP, 0.8 M betaine, 0.004% malachite green, 8 U/mL *Bst* 2.0 DNA Polymerase Warmstart (New England Biolabs), 1.6 µM of each FIP(-FAM) and BIP(-DIG) primer, 0.2 µM of each F3 and B3 primer, and 0.4 µM of LB primer (loop primer). Finally, 2 µL DNA were added into each tube as a template. The amplification occurred at 65 °C for 60 min in a heating block. In every experiment, positive control (DNA of *E. multilocularis*) and negative control (water) were included.

#### 4.2.4. Visualisation of the LAMP Reaction

Three different visualisation methods were used to assess the results of LAMP reactions. First, directly after the incubation time, visual judgment of the colour change was performed and documented. A positive reaction was indicated by different intensities of blue colour and in a negative reaction by a colour change from blue or green to colourless. Secondly, 3 µL of each LAMP product were subjected to gel electrophoresis in 1.5% agarose gels stained with Gel Red (Biotium, Fremont, CA, USA) and visualised with a UV transilluminator (BioRad, Hercules, CA, USA). A DNA ladder-like pattern represented a positive reaction while no ladder was a negative result. Finally, we used a lateral flow dipstick (HybriDetect 2T, Milenia Biotec, Giessen, Germany) to assess the LAMP reactions with the FIP and BIP labelled primers (Table 5). Briefly, 100 µL of citrate-phosphate-buffer (Milenia Biotec) were mixed with 10 µL of the LAMP reaction in a 1.5 mL tube. Then, the dipstick was inserted into each tube and the results were assessed after five to ten minutes. LAMP amplicons labelled with FAM and DIG bind to the anti-digoxigenin antibodies in the strip. The test band will appear on the dipstick, along with the control band, whereas in a negative LAMP reaction, only the control band is visible.

#### 4.2.5. Stability of the LAMP Master Mix

The stability of the LAMP master mix was assessed by preparing aliquots of 23 µL of a LAMP master mix (as described above) without DNA template in 0.2 mL tubes stored at 4 °C, over a period of six weeks [66]. Every week, two tubes were taken from the fridge and 2 µL of *E. multilocularis* DNA (1 ng/µL) were added to one tube and 2 µL of water to the second tube as negative control. The amplification and visualisation of the reaction occurred as explained above.

#### 4.2.6. Limit of Detection of LAMP

The limit of detection (No. of eggs) of the LAMP reaction with the primer set from Table 5 was established using eggs of *E*. *multilocularis* isolated from faeces of a naturally infected fox (as explained above). Eggs were aspirated individually with a micropipette under the microscope into 1.5 mL tubes. Four replicates of tubes containing ten, five, four, three, two, and one egg(s) were prepared in 30 µL of water. Egg suspensions were subjected to protocols 1A and 1B as explained below for DNA extraction.

### 4.3. DNA Extraction (Protocols 1A and 1B)

The methods presented basically aim to break the eggs of *E*. *multilocularis*, and subsequently lyse cells of the embryo to release DNA to be used directly in LAMP and multiplex-PCR without a further purification step. We use here the term “DNA extraction” to refer to these methods for treating taeniid eggs. For protocol 1A, egg suspensions were treated with 0.2 M NaOH (ratio 1:1) and incubated at 95 °C for 10 min in a heating block [26,41]. Tubes were centrifuged (quick spin) and then dilutions with Tris-HCl pH 8.3 (100 mM) were prepared (ratios 1:2, 1:10, 1:50, and 1:100). Finally, 2 µL of each dilution were used as a template for LAMP (developed in this study using primers from Table 5) and multiplex-PCR [15]; the dilution offering the most consistent result was chosen for the DNA extraction in the field study. For protocol 1B, egg suspensions were treated as in protocol 1A without dilution with Tris-HCl. Subsequently, 100 µL of Instagene Matrix (Bio-Rad) were added to each tube and incubated for another 15 min at 56 °C. After vortexing and centrifugation (12,000× *g* for 3 min), 2 µL of the supernatant were used directly as a template for LAMP (developed in this study) and multiplex-PCR [15].

### 4.4. Application of LAMP and Multiplex-PCR in Field Samples

#### 4.4.1. Fox Faecal Samples

In total, 63 faecal samples from foxes shot during the official hunting season (January–February) in the surroundings of Zurich (Switzerland) from 2018 until 2020 were used in this study. At necropsy, fox intestines were examined for the presence of *E. multilocularis* and other parasites based on the SCT method [4]. Faecal samples were collected from the rectum, deposited in 50 mL tubes, and stored at −80 °C for five days to inactivate taeniid eggs. Worm burden was assessed in 45 foxes necropsied in 2020 including 28 positive foxes and 17 negative animals to the presence of *E. multilocularis*. Two aliquots of two grams of faeces per sample were taken for independent isolation of taeniid eggs as previously described [10]. The material retained in the 21 µm filter was collected and carefully screened with an inverted microscope for the presence of taeniid eggs in 10 mL tubes with a flat side. Then, all tubes were centrifuged at 200× *g* for 10 min, the supernatant discarded and the sediment was transferred to a 1.5 mL tube for treatments with protocols 1A or 1B and subsequently use as a template for LAMP (from this study) and multiplex-PCR [15].

#### 4.4.2. Dog Faecal Samples

In total, we used 32 faecal samples from dogs collected as part of a large prevalence study for *Echinococcus* spp. in Kyrgyzstan between 2017 and 2018 [including a genetic characterisation of *E*. *multilocularis* [67] and identification by egg isolation + multiplex-PCR [15]]. In the study mentioned above, taeniid eggs were concentrated from dog faecal samples as previously described and DNA was isolated combining alkaline lysis and the QIAamp Kit [10,50]. Briefly, eggs were resuspended in 200 μL of distilled water and 25 μL KOH (1M) and 7 μL of DTT (1M) were added and incubated at 65 °C for 15 min. Afterwards, 60 μL 2M Tris-HCl pH 8.4 and 2 μL HCl (12.4N/≥37%) were pipetted into the tubes. Finally, 200 μL of Buffer AL (QIAamp Kit) and 20 μL of Proteinase K were added and incubated at 56 °C for 10 min. Then, 50 µL of Chelex solution (50%) were added and tubes were mixed in a rotator for 1 h at room temperature. After centrifugation, the supernatant (approximately 400 μL) was transferred to a new tube with 200 μL of ethanol (100%) and mixed; the content of each tube was then transferred into a Qiagen spin column. The protocol continues following the manufacturer instructions for the QIAamp DNA with two washes with buffer AW1 instead of one. DNA was eluted in 100 μL of 10 mM Tris-HCl, pH 8.3, and stored at −20 °C until use as a template in multiplex-PCR [15].

### 4.5. Application of LAMP and Multiplex-PCR in Whole Faeces (Protocols 2A and 2B)

Two aliquots of 400–500 mg were taken from faecal samples of 30 foxes infected with *E*. *multilocularis* and five from negative foxes from the same group of animals used for protocols 1A and 1B (diagnosed at necropsy/SCT). Dog faecal samples included 18 *E*. *multilocularis* positive and ten negative (diagnosed using egg isolation + multiplex-PCR). One aliquot was treated with the protocol 2A: 400 µL of NaOH solution (0.2 M) were added, mixed, and incubated at 95 °C for 10 min, then 1 mL of Instagene Matrix (Bio-Rad) was added and the tubes were kept at 56 °C for 15 min. The samples were vortexed, centrifuged at 12,000× *g* for 3 min, and 500 µL of the supernatant transferred to a new tube. From the supernatant, a 1:50 dilution with water was prepared and 2 µL were used as a template for LAMP and multiplex-PCR. The second aliquot was used for DNA isolation following the QIAamp Fast DNA Stool Mini Kit^®^ instructions for large stool volumes (protocol 2B).

### 4.6. Sensitivity and Specificity

The diagnostic sensitivities and specificities were calculated for protocol 1A and 1B by comparing the results of LAMP and multiplex-PCR with the SCT result in foxes and with the initial multiplex-PCR (from the prevalence study) result in the case of dogs.

## Figures and Tables

**Table 1:** Results of LAMP and multiplex-PCR ^1^ amplification of *E. multilocularis* using the product of two DNA extraction methods (protocols 1A and 1B) as a template from isolated taeniid eggs from 62 fox faecal samples and 32 dog faecal samples. In the case of foxes, the status of *E. multilocularis* infection was established at necropsy with the Sedimentation and Counting Test (SCT); for dogs, *E. multilocularis* infections status was established based on egg isolation/multiplex-PCR [15].

Host	Number of Animals Confirmed Positive (+) or Without (–) Detected *E*. *multilocularis* Infection (Method for Diagnosis)	Protocol 1A: NaOH/Dilution 1:50 with Tris-HCl			Protocol 1B: NaOH + InstaGene^TM^ Matrix		
		Microscopic Detection of Taeniid Eggs ^2^	LAMP Positive	Multiplex-PCR ^1^ Positive	Microscopic Detection of Taeniid Eggs ^2^	LAMP Positive	Multiplex-PCR ^1^ Positive
Foxes	44 + (necropsy/SCT)	30 positive	25	29	31 positive	28	27
		14 negative	1	3	13 negative	4	3
	18 − (necropsy/SCT)	5 positive	1	1	5 positive	1	1
		13 negative	0	0	13 negative	0	0
Dogs	22 + (multiplex-PCR ^1^)	20 positive	20	20	21 positive	21	21
		2 negative	0	0	1 negative	0	0
	10 − (multiplex-PCR ^1^)	10 negative	0	0	10 negative	0	0

^1^ as described in [15]; ^2^ independent egg isolation from two aliquots of two grams of faeces performed as previously described [10].

**Table 2:** Sensitivity and specificity values including confidence intervals in brackets for LAMP and multiplex-PCR for the detection of *E. multilocularis* using as template material treated with protocols 1A and 1B (see Table 1 for details) from foxes and dogs. Foxes were considered truly infected if intestinal stages of *E. multilocularis* were found in the intestine at necropsy/SCT. Dogs were considered as truly infected based on a positive multiplex-PCR [15] performed previously as part of a prevalence study in Kyrgyzstan.

	Foxes Protocol 1A		Foxes Protocol 1B		Dogs Protocol 1A		Dogs Protocol 1B	
	LAMP	Multiplex-PCR	LAMP	Multiplex-PCR	LAMP	Multiplex-PCR	LAMP	Multiplex-PCR
Sensitivity (IC 95%)	59 (43.2–73.6)	72.7 (57.2–85)	72.7 (57.2–85)	68.1 (52.4–81.3)	90.9 (70.8–98.8)	90.9 (70.8–98.8)	95.4 (77.1–99.8)	95.4 (77.1–99.8)
Specificity (IC 95%)	94.4 (72.7–99.8)	94.4 (72.7–99.8)	94.4 (72.7–99.8)	94.4 (72.7–99.8)	100 (69.1–100)	100 (69.1–100)	100 (69.1–100)	100 (69.1–100)

**Table 3:** Number of positive results for *E. multilocularis* in LAMP and multiplex-PCR using protocols 1A and 1B in faecal samples from foxes necropsied in 2020 related to the total worm burden for *E*. *multilocularis*.

		Protocol 1A: NaOH/Dilution 1:50 Tris-HCl		Protocol 1B: NaOH + InstaGene^TM^ Matrix	
# *E. multilocularis* at SCT	# Examined	LAMP Positive (Sensitivity)	Multiplex-PCR Positive (Sensitivity)	LAMP Positive (Sensitivity)	Multiplex-PCR Positive (Sensitivity)
0	17	1	1	1	1
2–20	9	1 (11.1%)	1 (11.1%)	2 (18.1%)	2 (18.1%)
21–100	7	4 (57.1%)	6 (85.7%)	6 (85.7%)	7 (100%)
>100	12	10 (83.3%)	12 (100%)	12 (100%)	12 (100%)

**Table 4:** Results of LAMP for *E. multilocularis* (from this study) and multiplex-PCR [15] using as a template the supernatant of 500 mg of fox (from Switzerland) and dog faeces (from Kyrgyzstan) treated with protocol 2A and from the DNA isolated with the QIAGEN stool kit (protocol 2B).

Host	Number of Animals Confirmed Positive (+) or Without (–) Detected *E*. *multilocularis infection* (Method for Diagnosis)	Protocol 2A: NaOH + InstaGene^TM^ Matrix		Protocol 2B: QIAamp DNA Stool Mini Kit	
		LAMP Positive	Multiplex-PCR ^1^ Positive	LAMP Positive	Multiplex-PCR ^1^ Positive
Foxes	30 + (necropsy/SCT)	10 ^2^	17	16 ^2^	20
	5 − (necropsy/SCT)	0	0	0	0
Dogs	18 + (multiplex-PCR)	2 ^2^	7	7	6
	10 − (multiplex-PCR)	0	0	0	0

^1^ as described in [15]; ^2^ all samples found to be positive in LAMP were also positive in multiplex-PCR.

**Table 5:** Primer set selected for the detection of *Echinococcus multilocularis* in a LAMP assay. For detection with the HybriDetect-Universal Lateral Flow Assay Kit (LFD), a FAM (6-fluorescein amidite) and DIG (digoxigenin) modifications were included in the Em-FIP and Em-BIP primers.

Primer	Sequence (5′–3′)	5′-Modification (LFD)
Em-F3	GCTTGTTGTTGTTTCCATTGA	-
Em-B3	ACAAAACCACCACCAACC	-
Em-FIP	TCCCTTTCAGACTCCCCATAATCA-TTTTTGGTGTGTGTGCTATG	FAM
Em-BIP	AGCGGTATATACTTTACGTGTTTGT-TCATTACAACAATCAACCATGA	DIG
Em-LB	TTGCTTGTGAGTATATAGTTGTATATGTGT	-

## Data Availability

Not applicable.

## Supplementary Materials

The following are available online at https://www.mdpi.com/article/10.3390/pathogens10070847/s1, Table S1. List with all the results from foxes positive and negative to intestinal stages of *E. multilocularis* diagnosed at necropsy and used in the present study. Isolated eggs were treated with protocol 1A and 1B. Results for multiplex PCR also shows if an amplicon corresponding to other cestodes (oc) including *Taenia* spp. was also amplified. Em: *Echinococcus multilocularis*.
